# Afferent innervation of the kidney: projections to and processing in the central nervous system

**DOI:** 10.3389/fphys.2025.1743631

**Published:** 2026-02-05

**Authors:** Brianna Dailey-Krempel, Louise C. Evans, John W. Osborn, Lucy Vulchanova

**Affiliations:** 1 Department of Neuroscience, University of Minnesota, Minneapolis, MN, United States; 2 Department of Surgery, University of Minnesota, Minneapolis, MN, United States

**Keywords:** afferent renal nerves, autonomic nervous system, dorsal root ganglia, interoception, renal innervation

## Abstract

FDA approval for catheter-based renal nerve ablation has sparked renewed interest in the role of renal nerves in hypertension and their potential contribution to other pathophysiologies. While the anti-hypertensive effects of catheter-based renal nerve ablation were thought to be due to the ablation of the sympathetic efferent nerves going to the kidney, preclinical hypertensive rodent studies and unexpected beneficial clinical outcomes have highlighted the renal afferent (sensory) nerves as a contributor to the pathogenesis of hypertension. Renal afferents are most abundant in the renal pelvis but also innervate the renal cortex. Their neurochemical and functional diversity remains to be fully elucidated. Tracing studies from the kidney to the central nervous system (CNS) have identified direct projections of spinal afferents with cell bodies in dorsal root ganglia to both the spinal cord and caudal brainstem. Few studies have suggested vagal innervation of the kidney with cell bodies in the nodose ganglia. The central processing of renal afferent input in brain autonomic circuits is not thoroughly understood. Regions involved in renal afferent input processing include the paraventricular nucleus of the hypothalamus and nucleus of the solitary tract which integrate peripheral sensory inputs with central homeostatic sensing regions including circumventricular organs. This review aims to summarize our current understanding of renal afferent anatomy including tracing and immunolabeling studies from the kidney to the CNS, the general mechanosensitive and chemosensitive subtypes in the kidney, and broadly discuss a potential pathway for the central processing of renal afferent input through autonomic and other homeostatic nuclei.

## 1 Introduction

A conventional view of renal afferent (sensory) projections from the kidney to the central nervous system formed primarily during the early 1980s–1990s. Initiated by electrophysiological studies, a link between renal afferent nerves and autonomic homeostatic centers was identified by measuring neuronal activity in the hypothalamus and medulla in response to afferent renal nerve stimulation (Ciriello and Calaresu, 1980; Calaresu and Ciriello, 1981). Anatomical studies in preclinical animal models (e.g., rats and cats) defined how renal afferents travel into the central nervous system using a variety of tracing methods. These studies found that renal afferents originate from thoracic and lumbar dorsal root ganglia (DRG) between T6-L3, and consist of terminations within the dorsal horn of the spinal cord (Ciriello and Calaresu, 1983; Kuo et al., 1983; Knuepfer et al., 1988) and direct projections to the caudal brainstem (Simon and Schramm, 1984; Wyss and Donovan, 1984; Knuepfer and Schramm, 1985; Gattone et al., 1986). Processing of afferent renal nerve input within the brain has yet to be fully described. This review aims to 1) organize prior tracing literature of afferent renal nerves into the central nervous system by comparing study methods and conclusions, 2) briefly discuss the mechanosensitive and chemosensitive properties, and 3) propose how afferent renal nerve responses to intrarenal stimuli may be processed through central autonomic circuits contributing to their involvement in homeostatic processes.

**FIGURE 1:**
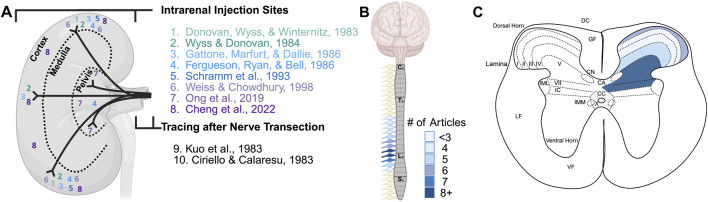
Summary of selected research articles that identified afferent innervation of the kidney. **(A)** Diagram of reported injection sites in the kidney or tracing after renal nerve transection from selected articles. **(B, C)** The number of articles (not specific to those in **(A)**) that reported tracer presence in dorsal root ganglia **(B)** and the location of terminations within the spinal cord **(C)**. Legend in **(B)** is for panels **(B, C)**. Abbreviations: C, cervical; T, thoracic; L, lumbar; S, sacral; DC, dorsal column; GF, gracile fasciculus; CN, Clarke’s nucleus; CA, central gray matter/gray commissure; CC, central canal; IML, interomediolateral cell column; IC, intermediate gray column/zone; IMM, interomediomedial cell column; LF, lateral fasciculus; VF, ventral fasciculus.

Relevant tracing studies are summarized in Table 1 for information on the range of DRG that contain renal afferent neurons and location of kidney injection sites. Table 2 includes studies that discuss parasympathetic or vagal innervation of the kidney. Table 3 shows reports on terminations within the spinal cord. Figure 1 depicts the kidney injection sites and selected findings in the DRG and spinal cord. The neurotracers, i.e., conventional and viral tracers, described in this review are summarized in Table 4 including a brief description of the method of uptake, considerations, and limitations for their use (Horikawa and Powell, 1986; Lanciego and Wouterlood, 2011, 2020; Nassi et al., 2015; Saleeba et al., 2019).

**TABLE 1:** Summary of studies that reported tracer uptake in dorsal root ganglia after intrarenal injection or transection of renal nerve.

Study (authors, year published)	Animal model	Method	Injection location	Ganglia range
Donovan et al. (1983)	Rat	Fluorescent dye	Renal cortex – single injection in rostral and caudal poles	T7-L3
Kuo et al. (1983)	Cat	HRP	Transected renal nerve	T12-L4
Ciriello and Calaresu (1983)	Rat	HRP	Transected renal nerve, injection in/around renal hilum	T8-L2
Wyss and Donovan (1984)	Rat	Fluorescent dye	Renal cortex – single, double, and triple injections in rostral-caudal poles	T8-L3
Gattone et al. (1986)	Rat	HRP-WGA	Renal cortex – 3 to 14 injections in anterior surface	T10-L2
Ferguson et al. (1986)	Rat	HRP	Perihilar fat, single injection in rostral or caudal pole	T11-L3
Schramm et al. (1993)	Rat	FluoroGold	Single injection into each pole (rostral-caudal)	T11-L2
Weiss and Chowdhury (1998)	Rat	PRV	Two injections into each pole (rostral-caudal)	T11-T12
Ong et al. (2019)	Mouse	WGA-Alexa	Renal hilum - three injections from rostral-caudal	T9-L2

Identification methods include tracing via fluorescent dyes (e.g., True Blue, Fast Blue), horseradish peroxidase (HRP) unconjugated or conjugated to wheat germ agglutinin (WGA), FluoroGold, WGA-conjugated to Alexa Fluorophore, adeno-associated virus (AAV) and pseudorabies virus (PRV). Abbreviations: T, thoracic; L, lumbar.

**TABLE 2:** Summary of studies that investigated parasympathetic and/or vagal innervation of the kidney.

Study (authors, year published)	Animal model	Method	Experiment and location	Conclusion
Norvell and Anderson (1983)	Mongrel dog; rat	HRP; HRP-WGA	Transected renal nerve, intrarenal injection of HRP-WGA	Negative
Donovan et al. (1983)	Rat	Fluorescent dye	Renal cortex – single injection in rostral and caudal poles	Negative
Calaresu et al. (1985)	Rat	Electrical stimulation	No injection, transection of cervical vagus, recording in renal nerve	Negative
Gattone et al. (1986)	Rat	HRP-WGA	Renal cortex – 3 to 14 injections in anterior surface	Positive
Zheng and Lawson (1994)	Rat	Fluorescent dye	Transected renal nerve, intrarenal injections	Negative
Ong et al. (2019)	Mouse	WGA-Alexa	Renal hilum - three injections from rostral-caudal	Negative
Cheng et al. (2022)	Mouse	AAV	Renal cortex - five injections from rostral-caudal	Positive

Descriptions of these studies are in Section 2.1.3. The study conclusion with supporting evidence or evidence against parasympathetic or vagal innervation of the kidney is reflected as positive or negative, respectively, in the “Conclusion” column. Identification methods include electrical stimulation and recording, and tracing via fluorescent dyes (e.g., True Blue, Fast Blue, FluoroGold), horseradish peroxidase (HRP) unconjugated or conjugated to wheat germ agglutinin (WGA), WGA-conjugated to Alexa Fluorophore, and adeno-associated virus (AAV). Abbreviations: T, thoracic; L, lumbar.

**TABLE 3:** Summary of studies that reported terminations in the spinal cord, including spinal cord level segments, lamina (Rexed reference), and other select spinal regions.

Study (authors, year published)	Animal model	Method	Spinal cord segment range	Lamina identified	Other spinal regions identified
Kuo et al. (1983)	Cat	HRP	T12-L4	V-VII	Dorsomedial central canal, Clarke’s column, IML
Ciriello and Calaresu (1983)	Rat	HRP	T6-L2	I, III-V	Dorsomedial central canal, Clarke’s column
Ammons (1986)	Cat	Electrophysiology	T12-L2	V, VII	
Ammons (1987)	Cat	Electrophysiology	T12-L2	V, VII I, V, VII	STT, SRT
Ammons (1988)	Cat	Electrophysiology	T12-L2	I, V, VII	LSRT
Knuepfer et al. (1988)	Rat	Electrophysiology	T9-L1	I, IV-VIII	
Schramm et al. (1993)	Rat	PRV	T5-T13	VII	IML, intermediate zone, IC, LF
Weiss and Chowdhury (1998)	Rat	PRV	T5-L3	I, II, IV, V, VII, X	Dorsomedial central canal, Clarke’s column
Sly et al. (1999)	Rat	PRV	T1-T8	Not examined	
Weiss et al. (2001)	Rat	PRV	T10-T13	I, III-V, X	Dorsomedial central canal, IML, IC, LF
Tang et al. (2004)	Rat	PRV	T6-L1	II-V, VII, X	IML, LF

Identification methods include tracing via horseradish peroxidase (HRP) or pseudorabies virus (PRV), and electrophysiology experiments. Abbreviations: IML, interomediolateral cell column; STT, spinothalamic tract; SRT, spinoreticular tract; LSRT, lateral spinoreticular tract; IC, intermediate gray column/zone; LF, lateral fasciculus.

**TABLE 4:** Comparison of neurotracing methods, including methods of uptake and considerations for use and interpretation.

	Tracer	Method of uptake	Considerations	Limitations
Conventional	Fluorescent dyes (i.e., Fast/True Blue, Nuclear Yellow, FluoroGold)	Fluid-phase endocytosis (pinocytosis)	Transport in 2–7 days; tissue processing not required for visualization; can be inefficient since passively taken up by terminals, but can be applied in larger volumes; not cytotoxic	• Non-selective uptake results in inability for increased selectivity for labeling of specific cell populations • Uptake by fibers-of-passage depending on injection site • Diffusion of tracer decreases ability for investigations of fibers near injection site • Consider direction of axonal transport for circuit analyses
	Horseradish peroxidase (HRP)		Transport in 2–7 days; requires tissue processing for visualization; can be inefficient since passively taken up by terminals, but can be applied in larger volumes or conjugated to WGA/CTB; not cytotoxic	
	DiI	Lipophilic – insertion into cell membrane	Transport in 2–7 days, also dependent on location of labeled target; results in more uniform labeling via lateral movement along cell membrane; tissue processing quenches fluorescence after lipid removal; not cytotoxic	
	Wheat germ agglutinin (WGA)	Receptor-mediated (adsorptive) endocytosis - binds to glycoproteins and glycolipids in cell membrane	Transport in 2–7 days; more efficient uptake due to binding of cell membrane; can be conjugated to fluorophore or HRP for visualization; not cytotoxic	
	Cholera toxin subunit B (CTB)		Transport in 2–7 days; can be conjugated to fluorophore or HRP for visualization; some quenching of signal when combined with IHC; not cytotoxic	
Viral	Pseudorabies virus (PRV)	Receptor-mediated endocytosis	Replication-competent; efficient transduction and labeling in 2–4 days; trans-synaptic labeling allows for tracing of entire circuits; non-selective uptake; cytotoxic to cells and animal after 4–6 days of labeling	• Circuit directionality requires time-course analysis of labeling
	Adeno-associated virus (AAV)	Capsid-mediated endocytosis	Replication-deficient; efficient transduction and labeling after 2–6 weeks; smaller injection volume compared to conventional tracers; different serotypes (e.g., capsids) for efficacious uptake in various tissue types, antero/retrograde and trans-synaptic labeling; in combination with transgenic mouse lines, a specific promotor or genetic restrictive systems (e.g., Cre-LoxP) allows for selective expression of transgenes in cell populations of interest; can use tissue processing to enhance reported signal; not cytotoxic	• Potential absence of cell-specific tropism

## 2 Tracing of renal afferent nerves

### 2.1 Renal afferent input to the central nervous system

#### 2.1.1 Cell bodies in DRG and terminations on dorsal horn spinal neurons

Anatomical evidence of renal afferent input to the central nervous system was initially identified using fluorescent dyes, e.g., True Blue and Fast Blue, injected directly into the renal cortex of rats by Donovan et al. (1983). True Blue and Fast Blue are passively taken up by the terminals of neurons, and the dye is retrogradely transported to the cell body. DRG neurons ipsilateral to the injected kidney were labeled from T8-L1 with highest abundance in the right DRG T11-T12 and left DRG T12-T13, and without any specific organization pattern within the ganglia. These results have since been repeatedly reproduced with other tracing methods to identify the distribution of renal afferent nerve input to the central nervous system and are described below.

Kuo et al. (1983) investigated the central projections of renal afferents to the spinal cord in cats using horseradish peroxidase (HRP) (Kuo et al., 1983). HRP is a retrograde tracer that is taken up by nerve terminals via endocytosis and transported to the cell body. HRP-labeled afferent axons were identified in the ipsilateral minor splanchnic nerves and cell bodies were found in DRG from T12-L4 in adult cats with a majority of neurons between L1-L3. Renal afferent spinal cord projections were further identified in kittens between T11-L6 with the greatest abundance of fibers in spinal levels L1 and L3. Renal afferent fibers were seen predominantly along the lateral border of the dorsal horn with collaterals in laminae V-VI. A smaller bundle also extended to laminae VII and ventrolaterally towards the intermediolateral cell column (IML). Some fibers transversed along the dorsomedial dorsal horn border and converged with branches from the lateral border near Clarke’s column. Few contralateral DRG were labeled, and some collateral projections were identified from the dorsomedial bundle to project contralaterally. This early study illustrates the intricate pattern of central terminations of renal afferent nerves into the spinal cord.

In the rat, Ciriello and Calaresu (1983) used the retrograde transport of HRP either by the placement of the dissected renal nerve into HRP or injection of HRP into and around the renal hilum (Ciriello and Calaresu, 1983). HRP-labeled DRG were identified in the left T8-L2 DRG and right T6-T13 DRG. Within the dorsal horn of the spinal cord, collaterals were identified in laminae I and III-V, with a majority of terminations in the mediodorsal aspect of the ipsilateral gray commissure, with no contralateral terminations.

Both Kuo and colleagues and Ciriello and Calaresu hypothesized that the collaterals around the IML and terminations near the dorsal gray commissure may be sites for renal afferent input onto preganglionic sympathetic neurons that are involved in spinal reno-renal reflex pathways (Ciriello and Calaresu, 1983; Kuo et al., 1983). Additionally, both studies described renal afferents as mostly unmyelinated (C) fibers with few being thinly myelinated (Aδ) based on the size of the corresponding DRG cell bodies. These descriptions were in agreement with prior studies showing these axon types within the kidney (Zimmermann, 1975; Barajas and Wang, 1978), and the suggestion that the two fiber types may correspond to different physiological response properties, i.e., input via mechanoreceptors by Aδ (Niijima, 1971; 1975) and chemoreceptors by C-fibers (Recordati et al., 1978; Recordati et al., 1980).

Complementing the anatomical descriptions by Kuo and colleagues and Ciriello and Calaresu (Ciriello and Calaresu, 1983; Kuo et al., 1983), several studies used electrical stimulation of renal nerves to identify the location of responsive spinal neurons. Ammons (1986) found that stimulated spinal neurons were mostly localized to laminae V and VII between T12-L2 and received input from Aδ and C-fibers in cats (Ammons, 1986). Input to spinal neurons in laminae V and VII is consistent with the identified renal afferent projections by Kuo et al. (1983). Renal nerve stimulation mostly excited these neurons, and few were inhibited. These spinal neurons had a range of response properties including latency to activation and magnitude. The combination of response properties and the potential integration of visceral signals with other somatic input on these spinal neurons reveal part of the complexity in spinal processing of renal afferent input.

Ammons (1987) also identified spinoreticular (SRT) and spinothalamic (STT) neurons that receive renal input between T12-L2 in cats (Ammons, 1987). SRT neurons were found in laminae I, V, VII and VIII with axons in the ventrolateral white matter. SRT neurons were separated into three groups based on their projection to the reticular formation in the caudal brainstem, i.e., ipsilateral, contralateral, or bilateral projecting. STT neurons were found in laminae I, V, and VII. SRT and STT neurons were responsive to both somatic and renal input. Additionally, Ammons (1988) observed renal and somatic input to lateral SRT (LSRT) spinal neurons via stimulation of the ventrolateral medulla. LSRT spinal neurons were found in laminae I, V and VII between T12-L2 in the cat (Ammons, 1988). These LSRT spinal neurons were responsive to renal mechanoreceptor activation from renal vein or ureteral occlusion via both Aδ and C-fibers.

Comparatively, Knuepfer and Schramm (1987) identified the electrophysiological properties of renal afferents by electrically stimulating renal afferents in the rat dorsal root between T9-L1 and recording responses in the renal nerve (Knuepfer and Schramm, 1987). The findings in this study agree with prior hypotheses of a combination of C-fibers and Aδ fibers identified in ultrastructural studies of the kidney (Zimmermann, 1975; Barajas and Wang, 1978), based on DRG cell body size (Ciriello and Calaresu, 1983; Kuo et al., 1983), and electrophysiological studies (Simon and Schramm, 1984; Ammons, 1986). No difference in the distribution of these fiber types was observed between T9 to L1, but most of the recordings of activation occurred between T11-T13.

Knuepfer et al. (1988) expanded on these findings by identifying spinal neurons in the dorsal horn that are responsive to electrical stimulation of the renal nerve in rats (Knuepfer et al., 1988). Neurons in laminae I, IV-VIII were responsive to renal nerve stimulation, with most responses in laminae IV and V. Many of these neurons were also responsive to cutaneous stimulation, however, a couple of neurons in lamina I were selectively responsive to renal nerve stimulation, but not cutaneous stimulation.

A more recent study by Ong et al. (2019) aimed to use a neurogenic hypertensive model to elucidate the role and anatomy of the renal afferents in the mouse (Ong et al., 2019). The authors used wheat germ agglutinin (WGA) conjugated to an Alexa Fluorophore injected into the left or right renal hilum to identify the distribution of renal afferent cell bodies in the DRG and nodose ganglia. WGA is a lectin that can bind to glycoproteins and glycolipids, and is transported into neurons via receptor-mediated endocytosis and then transported to the cell body. There was observed labeling from the left kidney between T9-L2 and right kidney between T7-L3, with a majority of cells in T12-L2 or T11-L2, respectively. The authors describe their results as one of the first to anatomically analyze the distribution of renal afferents in the mouse, compared to studies primarily done in larger animal models, e.g., rat, cat, and dog.

In comparison to the well described pathway of somatosensory information from the periphery to the central nervous system (including innocuous and noxious stimuli), the notable extent of input from renal afferents onto many spinal laminae suggests an intricate network for supraspinal and intraspinal processing. Renal input to laminae V and VII in the rat and cat overlap with other visceral organ inputs, suggesting convergence of visceral sensory information within these regions (Ammons, 1986, 1987, 1988; Knuepfer et al., 1988). This convergence may result in renal-mediated reflex responses, e.g., reno-renal reflexes, or provide an integration point for visceral sensory information for other interoceptive processes. The STT pathway is the canonical pain transmission pathway, and the convergence of somatic and renal stimuli on STT neurons in the spinal cord may transmit renal pain signals to higher order centers and contribute to referred pain. Since the reticular formation is important for modifying awareness, renal input to both STT and L/SRT neurons provides a circuit for renal afferent signals to be integrated with other visceral inputs for autonomic regulation, e.g., LSRT input to ventrolateral medulla.

#### 2.1.2 Direct projections and terminations in the caudal brainstem

Myelinated renal afferents were found to project directly through the dorsal column of the spinal cord to terminate in both the gracile nucleus and the nucleus of the solitary tract (NTS) in the caudal brainstem in Simon and Schramm’s 1984 study using electrical stimulation (Simon and Schramm, 1984). These myelinated renal afferent fibers were identified by electrically stimulating axons in the upper cervical spinal cord or the caudal brainstem, while recording in the renal nerve via a “collision” test approach allowing for the stimulation and recording of the same axon at two different sites.

This direct projection from the kidney to the brainstem was confirmed anatomically by Wyss and Donovan (1984) using a double fluorescent dye technique (Wyss and Donovan, 1984). Fast Blue was injected into the renal cortex, while Nuclear Yellow was injected into the caudal brainstem. DRG neurons that were double labeled blue in the cytoplasm and yellow in the nucleus were classified as kidney-innervating DRG neurons that had a direct monosynaptic projection to the caudal brainstem. Double labeled DRG neurons accounted for approximately 8% of all labeled renal afferent neurons and were ipsilateral to the injected kidney between DRG T8-L2, with most in T11.

Knuepfer and Schramm (1985) found that reno-bulbar spinal afferent fibers, i.e., directly projecting from the kidney to the caudal brainstem, were responsive to increases in intrarenal pressure, but not chemical stimuli, and had receptive fields within the renal parenchyma (Knuepfer and Schramm, 1985). This finding suggests that direct input from the kidney to the brainstem provides information on changes in intrarenal pressure, including perfusion and filtration pressure, rather than changes in the chemical microenvironment. This direct circuit is likely important in acute changes in renal blood flow and regulation of systemic arterial pressure.

#### 2.1.3 Cell bodies in the nodose ganglion

Norvell and Anderson (1983) investigated whether the kidney has parasympathetic innervation, but did not observe any supporting evidence (Norvell and Anderson, 1983). In the initial identification of renal afferent cell bodies in DRG using fluorescent dyes, Donovan and colleagues also reported labeling patterns in the nodose (inferior vagal) ganglia, which contains vagal afferent cell bodies, and dorsal motor nucleus of the vagus nerve (DMX), however, this labeling was interpreted as nonspecific based on control injection experiments (Donovan et al., 1983). The control experiments included renal denervation and dye injection into the intraperitoneal space and tail vein. Control animals had a lack of dye labeling in DRG in all control experiments, but labeling in the nodose ganglia and DMX persisted, suggesting the dye was taken up by free nerve endings in the vasculature and peritoneal lining.

Calaresu and colleagues (1985) investigated the potential of vagal innervation of the kidney using electrophysiology by stimulating the cervical segment of the vagus nerve and recording in the renal nerve (Calaresu et al., 1985). Evoked responses were recorded in both the contralateral and ipsilateral renal nerves. These responses were attenuated by ganglionic blockade and the transection or cooling of the splanchnic nerve, indicating the evoked renal nerve activity was sympathetic in origin. The authors concluded the stimulation-activated vagal afferents likely communicate, directly or indirectly, with sympathetic postganglionic neurons, resulting in sympathetic efferent output to the kidney that could represent a vago-renal reflex pathway. The authors reported that stimulation of the abdominal vagus did not result in evoked responses in the renal nerve, suggesting the vagal afferents stimulated in the cervical vagus are likely from cervical or thoracic structures, e.g., afferents from cardiopulmonary structures. In the context of the concurrent research on spinal afferents described above and overall lack of evidence for parasympathetic efferent innervation of the kidney (Norvell and Anderson, 1983), these findings had directed research efforts predominately towards sympathetic efferent and spinal afferent innervation in the following decades.

Gattone et al. (1986) reported similar ipsilateral DRG labeling between T9-L2, but noted a higher abundance of labeling bilaterally in the nodose ganglia using HRP conjugated to WGA (Gattone et al., 1986). From these novel findings, they concluded that the kidney is primarily innervated by vagal afferents in the nodose ganglion, however, few reports have been able to identify renal afferent labeling in the nodose ganglion, especially to the extent observed in this study. These researchers performed three control experiments: 1) application of HRP-WGA to the outer surface of the kidney (intraperitoneal application), 2) unilateral cervical vagus transection followed by a tail vein injection of HRP-WGA or injection into the inferior vena cava below the left renal vein, and 3) renal denervation. Labeled cells were observed in the DRG and nodose ganglia after the intraperitoneal application and venous injections of the tracer. There was a reduction in labeled cells in DRG and nodose ganglia after renal denervation. These findings are similar to those described from the control experiments by Donovan et al. (1983), and adds additional evidence for tracer uptake from free nerve endings in the peritoneal lining and vasculature. A difference between these two studies are the tracers that were used, i.e., fluorescent dye versus HRP-WGA that likely have different uptake efficiencies even though by similar mechanisms. Nonetheless, the inability to recapitulate these findings has further highlighted the abundance of evidence for sympathetic and spinal afferent innervation of the kidney and lack of support for the potential of vagal afferent innervation.

Few additional studies have looked at the nodose ganglia due to the focus on sympathetic and spinal afferent innervation. Zheng and colleagues looked for labeling in their rat study to characterize renal afferent subtypes by combining immunostaining with Fast Blue and FluoroGold tracing (Zheng and Lawson, 1994). FluoroGold is taken up by neuron terminals via endocytosis and is transported to the cell body. Ong and colleagues also looked at the nodose ganglia in their study on mouse renal afferents (Ong et al., 2019). However, neither study reported convincing labeling in the nodose ganglia.

The possibility of parasympathetic innervation of the kidney was raised again recently in a report by Cheng and colleagues (2022), where a retrograde adeno-associated virus (AAV) was used in combination with a transgenic mouse line (Cheng et al., 2022). In comparison to other neurotracing techniques, AAVs offer more flexibility and specificity for a particular tracing strategy through various viral serotypes with different efficacies in a number of tissues (Suarez-Amaran et al., 2025). AAVs can be combined with transgene cassettes creating customizable viruses for an experiment, e.g., transgene inserts for generating loss/gain-of-function genotypes, conditional expression, and fluorescent proteins for visualization. Using an AAV retrograde virus that expresses Cre under the human synapsin promoter (AAV2-hsyn-Cre) in Ai14 mice that express the fluorescent reporter tdTomato in a Cre-dependent manner, the authors injected the left kidney to label kidney-innervating neurons. Some cell bodies in the nodose ganglia were positively labeled for tdTomato indicating vagal afferents innervate the kidney. Immunostaining for vesicular glutamate transporter 2 (VGluT2) in the nodose ganglia identified the tdTomato+ cells as glutamatergic and showed projections and terminations in the NTS, suggesting an excitatory vagal afferent projection pathway from the kidney to the NTS.

Compared to prior investigations into the parasympathetic innervation of the kidney, Cheng and colleagues’ report contributes strong tracing evidence supporting parasympathetic efferent and vagal afferent innervation of the kidney. The visualization of parasympathetic innervation may now be possible with mouse transgenic and AAV tools that allow inducible, time-dependent, or permanent labeling. These findings require validation by additional investigations through similar techniques for labeling parasympathetic and vagal renal fibers. While labeled cells were identified in the nodose ganglia, i.e., vagal afferent neurons, from retrograde tracer injections in the kidney, these researchers did not describe the extent of vagal innervation within the kidney. This study described the parasympathetic efferent innervation of the kidney and the potential roles of these efferent fibers in kidney function in greater detail, and is not the focus of this review. The observed branching patterns of efferent fibers in the kidney were similar to those observed by renal sympathetic and spinal afferent fibers suggesting a similar distribution of vagal afferent fibers may be present. A summary of the evidence supporting (i.e., positive) and against (i.e., negative) the presence of parasympathetic efferent and vagal afferent fibers in the kidney is in Table 2. While the presence of vagal afferent renal innervation remains inconclusive, the evidence presented by Cheng and colleagues strongly suggests the presence of parasympathetic efferent innervation.

Future investigations will need to focus on identifying if vagal afferent fibers enter the renal cortex to the extent of innervation known for sympathetic and spinal afferent fibers. These findings raise many questions as to the role of parasympathetic efferents and vagal afferents in kidney function. Based on the initial findings, and lack of other evidence of parasympathetic efferent fibers in the renal cortex, these fibers may have a role in the overall perfusion of the kidney, rather than modulate or influence the function of specific structures, e.g., renal pelvic wall or nephrons. Renal vagal afferents may similarly preferentially innervate the renal vasculature and convey information on the chemical microenvironment and perfusion pressure. Similar to the direct renal spinal afferent pathway, a projection from the kidney via the vagus nerve directly to the NTS may provide an additional pathway for quick input to autonomic nuclei for homeostatic regulation.

### 2.2 Neurochemical subtypes of renal afferents

Neurochemical analysis of renal afferents initially focused on the colocalization of a tracer with labeling for the afferent neuron markers substance P (SP) and calcitonin gene-related peptide (CGRP). The proportions of CGRP (80%–90%) and SP (20%–24%) immunolabeled kidney-innervating DRG neurons were similar across three different species (cat, rat, and guinea pig) (Kuo et al., 1984; Su et al., 1986; Burg et al., 1994). In the rat, T9-L1 DRG contained a range of 53–85 dye-labeled neurons with 85% CGRP+ and similar proportions of SP+ and CGRP+/SP+ (Burg et al., 1994). These results agreed with evidence for colocalization of CGRP and SP labeling of nerve fibers in the kidney (Knight et al., 1991), suggesting that SP is co-expressed in a subset of CGRP+ neurons. Notably, 15% of dye-labeled cells were not labeled for either SP or CGRP, indicating the existence of other neurochemical subtypes of renal afferents (Burg et al., 1994).

Zheng and Lawson (1994) used immunolabeling for neurofilament 200 (RT97) and peripherin in rat DRG to distinguish between myelinated and unmyelinated dye-labeled renal afferents, respectively (Zheng and Lawson, 1994). A majority (79%) were classified as unmyelinated C-fibers and the remaining classified as thinly myelinated Aδ fibers. This classification agreed with prior ultrastructural analyses (Zimmermann, 1975; Barajas and Wang, 1978), tracing (Ciriello and Calaresu, 1983; Kuo et al., 1983), and electrophysiological analyses (Simon and Schramm, 1984; Ammons, 1986; Knuepfer and Schramm, 1987; Ammons, 1988). Retrogradely labeled Aδ and C-fiber neurons both expressed CGRP, but SP appeared to be exclusively present in C-fibers. This study further highlighted the neurochemical diversity of renal afferent DRG neurons, providing strong evidence for subtypes that likely contribute to different sensory functions within the kidney.

Ditting et al. (2009) investigated the role of peptidergic renal afferent neurons (SP or CGRP-expressing) that co-express the transient receptor potential vanilloid type 1 (TRPV1) channel in kidney inflammation during hypertension in rats (Ditting et al., 2009). During hypertension, renal inflammation and injury increase the acidity within the renal parenchyma, and CGRP has been shown to have a nephroprotective effect. Retrogradely labeled renal afferents had enhanced sensitivity to acidic stimuli that was blocked by a TRPV1 antagonist, suggesting a TRPV1-mediated acid sensing mechanism for peripheral CGRP release from primary afferent neurons. This was supported by the colocalization of TRPV1+ and CGRP+ immunolabeling in nerve fibers, which were most dense within the renal pelvis, but were also associated with tubules, blood vessels and glomeruli in the renal cortex. Recently, TRPV1 immunolabeling was demonstrated in ∼85% of kidney-innervating DRG neurons in the rat (Stocker and Sullivan, 2023), but the overlap of CGRP and TRPV1 expression has not been examined directly. It is notable that the proportions of CGRP+ and TRPV1+ mouse renal DRG neurons have not been reported, and previous work raises the possibility of species differences (Price and Flores, 2007).

Taken together, the above studies suggest functional diversity of renal afferents based on the presence or absence of different neuropeptides (CGRP+ only, CGRP+/SP+, and CGRP-/SP-) in unmyelinated C-fiber and lightly myelinated Aδ neurons as well as the expression of molecular transducers such as TRPV1. Comprehensive profiling of the different subtypes of renal afferents will potentially lead to ways to selectively target them and identify their functional roles, benefitting the overall understanding of renal interoception.

### 2.3 Distribution of renal afferent innervation within the kidney

Understanding of the distribution of afferent nerves within the kidney is largely built on immunohistochemical visualization of neuropeptides. Reinecke and Forssmann (1988) compared the distribution of markers for efferent (e.g., neuropeptide Y–NPY, neurotensin, vasoactive intestinal peptide–VIP) and afferent (e.g., CGRP, SP, somatostatin) renal nerves in kidney slices of several mammals. They reported a perivascular plexus containing NPY+, neurotensin+ and VIP+ fibers along the branches of the renal arterial tree. Neurotensin+ fibers were also observed near the juxtaglomerular apparatus (JGA) in the renal cortex. CGRP+ and SP+ fibers were observed close to renal hilum arterial branches and the JGA. Somatostatin+ fibers were seen within the smooth muscle of renal hilum arterial branches; however, another report did not observe any somatostatin+ fibers attributing the lack of labeling to potential detection limits or lower abundance (Burg et al., 1994). Quantitative analysis of immunostained fibers across species (i.e., Tupaia, dog, pig, guinea pig, and rat) revealed that the overall distribution of afferent and efferent fiber markers appeared similar across species, but that the pattern of labeling and fiber abundance for individual neuropeptides was distinct. These observations suggest potential species-specific contributions of different subtypes of afferent nerves in renal function. Interestingly, the authors performed a vagotomy and chemical denervation via intraperitoneal injection of 6-hydroxy-dopamine and found that very few of the renal nerves degenerated after vagotomy while most degenerated after chemical denervation, likely due to the targeting of catecholaminergic (efferent) fibers. Ferguson and Bell (1988) investigated the afferent innervation of the kidney in rats and found SP and CGRP labeled axons only in the renal pelvis (Ferguson and Bell, 1985; 1988). Similar observations were made by others (Reinecke and Forssmann, 1988; Knight et al., 1991), in contrast to the consistent visualization of sympathetic nerve labeling throughout all regions of the kidney. These studies strongly suggested that renal afferents appear to solely innervate the renal pelvis, and not the renal cortex, and further supported the predominance of sympathetic and spinal afferent innervation of the kidney with limited or no evidence for parasympathetic efferent or vagal afferent innervation.

Unlike the immunolabeling-based analyses described above, Marfurt and Echtenkamp (1991) used HRP-WGA injected into rat T12-L1 DRG to anterogradely label renal afferents and identify their regional distribution throughout the kidney (Marfurt and Echtenkamp, 1991). When injected into the kidney, WGA binds to surface glycoprotein and glycolipid complexes of cells, resulting in a robust injection site labeling that prevents detection of labeled nerve fibers. In contrast, anterograde tracing by injecting directly into the DRG allows tracer uptake at the cell soma and transport to the peripheral endings for the visualization of fiber distribution throughout the kidney. A direct DRG injection specifically labels only sensory neurons, resulting in a higher certainty of visualizing only afferent innervation. Anterogradely-labeled renal afferents appeared to enter the kidney via two routes: 1) along the renal artery branching extensively within the renal pelvis and following to the interlobar arteries and calyces, and 2) via smaller bundles observed to innervate the proximal portion of the ureter, entering the renal pelvis through the ureteropelvic junction. Consistent with other reports, afferent innervation was most abundant in the renal pelvis (Ferguson et al., 1986; Reinecke and Forssmann, 1988; Knight et al., 1991; Marfurt and Echtenkamp, 1991; Ditting et al., 2009). Renal pelvic afferent fibers wrapped circumferentially and terminated as free nerve endings in the smooth muscle and epithelial layers. Afferent innervation of the arterial tree was most dense distally, e.g., at interlobar and arcuate arteries, in contrast to the dense innervation of the proximal venous tree within the renal hilum. These observations agreed with other studies that showed afferent innervation at all levels of the renal vasculature (Reinecke and Forssmann, 1988; Knight et al., 1991; Marfurt and Echtenkamp, 1991). In the context of earlier reports, the authors speculated that the circumferential innervation within the renal pelvis may transduce changes in pelvic pressure (mechanosensitive) (Niijima, 1971; Uchida et al., 1971), while free nerve endings throughout the pelvic wall may respond to urine concentration and those innervating the vasculature respond to ischemia (chemosensitive) (Recordati et al., 1978; Recordati et al., 1980). The renal cortex was sparsely innervated with more fibers near the renal tubules than near glomeruli and occasional single fibers seen close beneath the renal capsule. Taken together, the observations in this study established that, while renal afferent nerves are more abundant in the pelvis and along the renal vasculature, they are also present in the cortex near tubules and glomeruli.

Evidence of the presence of cortical renal afferent nerves in proximity to different parts of the nephron has been relatively sparse (Reinecke and Forssmann, 1988; Marfurt and Echtenkamp, 1991; Ditting et al., 2009). Although in some of the tracing studies successful retrograde labeling was reported when tracer injections were restricted to the renal cortex (Donovan et al., 1983; Ferguson and Bell, 1988), most immunohistochemical studies only reported observations of renal afferents in the renal pelvis. Furthermore, DRG neurons were not consistently labeled by tracer injections in the renal cortex. For example, Ferguson et al. (1986) described HRP+ labeling in DRG from injections into the renal perihilar fat, but lack of DRG labeling from renal cortical injections (Ferguson et al., 1986). Overall, these conflicting results have left the anatomical understanding of renal cortical afferents understudied compared to the renal pelvic counterparts, and raises two questions: 1) are renal cortical afferents anatomically and physiologically different than renal pelvic afferents, and 2) if so, what is their role within the renal cortex.

In a recent report, Tyshynsky and colleagues (2023) used the transgenic mouse cross TRPV1-Cre x Ai14 to endogenously label TRPV1-lineage cells with tdTomato and immunostaining for CGRP to visualize renal cortical afferents (Tyshynsky et al., 2023). A robust analysis pipeline using a scoring method that categorized tdTomato+ and CGRP+ fibers in terms of their closeness to the glomerular portion of the nephron and glomerular depth within the renal cortex was combined with tissue clearing and high-resolution imaging to render 2D and 3D images of labeled fibers near glomeruli. The results demonstrated that ∼45% of analyzed glomeruli had a closely apposed CGRP+ axon and ∼58% had a tdTomato+ axon, and these associations were most prevalent among juxtamedullary compared to superficial and midcortical glomeruli. The cleared tissue analysis allowed for more precise quantification of the distance between the axons and the glomerulus. Of the analyzed glomeruli, ∼66% had a minimum distance less than 3 µm, and midcortical glomeruli had the longest distance at ∼4.1 µm compared to superficial ∼1.7 µm and juxtaglomerular ∼3.7 µm. Some axons were observed near glomeruli for 10–11 µm of their length, and in some cases appeared directly adjacent to the Bowman’s capsule of the glomerulus. Interestingly, the branching pattern of CGRP+ axons as they travel along the arterial vasculature to afferent arterioles suggested that one axon may contact multiple glomeruli. The functional role of these renal cortical afferents is unknown, but based on their anatomical relationship to glomeruli, suggest they may be involved in monitoring glomerular filtration pressure or the chemical microenvironment of the renal parenchyma around the nephron.

With these new anatomical insights, additional studies are required to further delineate the distinctions between renal cortical and pelvic afferents, highlighting an exciting upcoming area of basic science research. The distinct structural features of the renal cortex and pelvis and their functional specializations in urine production suggest that the encoding of relevant mechanical and chemical stimuli necessitates distinct subtypes of renal afferent nerves. However, the established renal afferent markers (e.g., CGRP, SP, TRPV1) are present in both pelvic and cortical afferents (Reinecke and Forssmann, 1988; Ditting et al., 2009; Tyshynsky et al., 2023). State-of-the-art viral tracing and genetic tools offer new approaches for identifying more selective subtype markers and investigating the regional specialization of renal afferents.

### 2.4 Renal afferent responses to mechanical and chemical stimuli

#### 2.4.1 Mechanosensation

Mechanosensitive renal afferents respond to intrarenal pressure changes (Ueda et al., 1967; Uchida et al., 1971; Niijima, 1971; Moss, 1989; Kopp, 2015). Ueda et al. (1967) measured efferent and afferent renal nerve activity while manipulating intrarenal pressure in mongrel dogs (Ueda et al., 1967). They reported that renal afferent nerve activity increased with compression of the kidney and renal vein occlusion (increase in perfusion pressure), but decreased with renal artery occlusion (decrease in perfusion pressure). The authors noted the opposite nerve activity in renal efferent nerves in the same settings, which was consistent with their prior report on a kidney-derived pressor reflex (Ueda et al., 1964). This work was expanded to describe two subtypes of renal mechanoreceptors, Type A and Type B, with different electrophysiological responses to changes in intrarenal pressure (Uchida et al., 1971). Type A renal afferents were smaller fibers, had a low voltage threshold, spontaneous discharge, and slow adapting response properties. Their firing rate was closely related to the waves of intrarenal pressure changes indicating responses within a physiological range. Type B renal afferents were larger fibers, had a higher voltage threshold, did not have a spontaneous discharge, and were fast adapting. Type B were noted to fire action potentials during changes in intrarenal pressure outside of the physiological range, potentially suggesting a response to more pathophysiological conditions. Both Type A and B afferents were suggested to be sensitive to intrarenal rather than intravascular pressure changes based on renal artery and vein occlusion. The authors speculated that these nerve endings may be within the renal interstitial space responding to changes in interstitial pressure and the differences in their properties could be due to their distinct locations within the kidney.

Niijima (1971) described similar responses of mechanosensitive renal afferents in the rabbit that was consistent with the Type A receptor subtype (Niijima, 1971). The main descriptive difference between these two studies was that Niijima localized the Type A-like renal afferents to perfusion pressure changes in the renal arterial wall compared to the interstitial space as hypothesized by Uchida et al. (1971). There was no observed firing with increased pressure in the renal vein. The Type A-like afferents were slow adapting with a gradual increase with increased perfusion pressure or mechanical indentation on the surface of the kidney, and spontaneously fired during physiological perfusion pressure and systemic blood pressure. Niijima compared the observed mechanosensitive renal afferents to baroreceptors in the carotid body with their similarity in placement within the arterial wall, but difference in their lack of synchronicity with heart rate. These afferents were also not as responsive to anoxia, so do not likely convey any chemoreceptive information from within the arterial vasculature.

Niijima (1975) further localized renal mechanoreceptors in the rabbit to all parts of the kidney and identified most as C-fibers with very few Aδ fibers (Niijima, 1975). A pointed pressure stimulus across the surface and internal regions of the kidney resulted in a burst of action potentials. Mechanical distention with forceps separating the renal pelvic walls also resulted in a burst discharge. These findings suggest a uniform distribution of mechanoreceptors throughout the different regions and tissues of the kidney. The receptive field of these mechanoreceptors were estimated to be spot-like with 1–2 mm diameters. Receptive fields appeared to be innervated by single fibers with responses of that nerve localized to only that receptive field. Mechanoreceptors were described in two main parts of the kidney: the parenchyma and the pelvic wall. Application of a pressure stimulus on the surface of the kidney activated mechanoreceptors deeper than the renal cortex. Niijima also concluded that mechanoreceptors in the kidney are responsive to changes in intrarenal perfusion pressure and distention of the pelvic wall, which agrees with the findings described above in mongrel dogs. There was a brief mention of mechanosensitive renal pelvic afferents in potentially relaying kidney-pain-like stimuli, however, evidence for renal afferents that convey a pain-like signal has not been individually identified and likely relies on a combination of stimuli input from the kidney.

#### 2.4.2 Chemosensation

Chemosensitive renal afferents haven been generally classified into two categories: R1 or R2 (Recordati et al., 1978; Recordati et al., 1980, 1981; Moss, 1989; Kopp, 2015). In the rat, R1 chemoreceptor renal afferents responded to complete renal ischemia, e.g., renal artery (RAO) or vein occlusion (RVO), systemic asphyxia, and severe hypotension (Recordati et al., 1978). R1 renal afferent responses were characterized by having no spontaneous activity, and firing in a train of impulses 30 s after stimulus. Based on their greater responsiveness to RVO compared to RAO, it was postulated that R1 chemoreceptive afferents are more active in severe cases of hypotension (i.e., <40 mmHg) rather than systemic changes in blood pressure or renal perfusion pressure. This was supported by lack of R1 responsiveness to changes in renal pelvic pressure suggesting the sensitivity of R1 afferents to systemic hypotension was due to renal ischemia. Based on the conduction velocity of these axons, the authors concluded that R1 chemoreceptive afferents are C-fibers.

Recordati et al. (1978) reported a separate population of renal afferents that had an increase in firing during the backflow of urine into the renal pelvis, and were not responsive to changes in pelvic pressure or ischemia. The authors characterized the response properties of R2 renal afferents and how they differed from their R1 counterparts in their 1980 article (Recordati et al., 1980). R2 chemoreceptor renal afferents had a low tonic discharge rate that increased with the backflow of urine from the ureter into the renal pelvis, but not with isotonic saline suggesting they were not responsive to the change in pelvic pressure. The researchers tested solutions with different ionic compositions and osmolality, and diuretic versus non-diuretic urine by measuring before, during and after a 5% volume expansion with isotonic saline. R2 afferents were increasingly responsive to higher concentrations of NaCl (3%–5%) and mannitol compared to lower concentrations of NaCl (1.5%) or urea, and maximally excited by an isotonic concentration of KCl compared to higher concentrations. They found that firing decreased with the isotonic saline expansion while the urine flow rate and sodium concentration increased, and potassium and urea concentrations decreased inducing a diuretic state (reduced solute concentration). Infusion of non-diuretic urine (normal concentration) increased the firing rate of R2 afferents. During RAO, R2 afferents had a quick discharge rate, but then maintained a steady firing, which is in contrast to the quick increase and then steady decrease in firing of R1 afferents. The authors discuss that the increased firing of R2 afferents during occlusion is likely due to the change in solute concentration that is induced by the occlusion such as localized hypoxia within the renal parenchyma and resulting cell responses to the hypoxic state. The authors further discuss the location of renal afferents within the renal cortex versus pelvis, how the diffusion of filtered solutes may contact afferent nerve endings of the afferents in the different regions, and how the renal pelvic distention may have a role in modifying epithelial permeability, e.g., the permeability of the epithelium between the renal pelvis and medulla into the renal interstitium versus the permeability of these solutes across the pelvic wall epithelium. These findings were complemented by a third report from the same authors in 1981 that summarized the roles of R1 and R2 chemoreceptive renal afferents (Recordati et al., 1981).

### 2.5 Trans-synaptic tracing of renal nerves with pseudorabies virus (PRV)

PRV has been used in rats to trans-synaptically identify the neurons involved in sympathetic outflow from the brainstem to the kidney (Schramm et al., 1993; Weiss and Chowdhury, 1998; Sly et al., 1999). PRV is a retrograde virus that crosses the synapse of connected neurons allowing for the tracing of entire circuits, i.e., more neurons are labeled with increased time post-injection, followed by immunolabeling to visualize the tracing. PRV is more rapidly taken up by sympathetic efferent nerves in the kidney, rather than afferent nerves, resulting in initial labeling of postganglionic neurons in chain ganglia and then preganglionic neurons in the IML, followed by some labeling of renal afferent circuitry via projections between the IML and dorsal horn in the spinal cord. These studies describe the pattern of labeling in terms of “order”, not the way they transmit information. Since PRV is a retrograde virus in the efferent component of the circuitry, neuronal signals would pass from the fourth order neuron to the first in descending order, but the reverse for the afferent component of the circuitry.

Different strains of PRV (Weiss and Chowdhury, 1998) and larger volumes (Sly et al., 1999) injected in the rat renal cortex followed by analysis at multiple post-injection (PI) time points have been used to compare and confirm structures in the renal afferent pathways identified with other tracing methods, e.g., fluorescent dyes and HRP (Weiss and Chowdhury, 1998; Sly et al., 1999). The wildtype PRV strain, Becker, had the highest labeling at 2 days PI compared to two attenuated PRV strains, Bartha and SyntroVet, that required 3–4 days for adequate labeling (Weiss and Chowdhury, 1998). Results between these three strains were consistent with initially reported findings (Schramm et al., 1993). Sly and colleagues described four orders of neurons, e.g., the retrograde path or order of labeling, compared to three orders previously described (Schramm et al., 1993; Weiss and Chowdhury, 1998). This labeling reached cortical brain regions providing insight into potential top-down pathways that regulate kidney function. The analysis of labeling in the days PI provided the progression of PRV labeling across synapses suggesting a potential order in which afferent information from the kidney is transmitted as well as the supraspinal descending pathway for sympathetic outflow.

Broadly, PRV-labeled neurons were identified 2 days PI in postganglionic sympathetic efferents (e.g., in sympathetic paravertebral, suprarenal, celiac, and superior mesenteric ganglia; first order) and preganglionic neurons in the IML and intermediate gray zone (IC) (second order). Days 2–3 PI resulted in labeled neurons in the contralateral IML and ipsilateral dorsal and ventral horns. Third order labeled neurons were visible 3 days PI and were mainly localized to caudal brainstem sympathetic premotor nuclei, i.e., medullary raphe nuclei, rostral ventrolateral medulla (RVLM), rostral ventromedial medulla (RVMM), A5 cell group, and the paraventricular nucleus of the hypothalamus (PVN). At 3 days PI, labeling in the PVN was limited to the dorsal (majority) and ventral parvocellular subdivisions, and nearly equal labeling 4 days PI with additional labeling in the dorsomedial parvocellular and lateral magnocellular subdivisions. Day 4 PI showed little labeling in the nucleus of the solitary tract (NTS), area postrema, periaqueductal gray, locus coeruleus, and subcoeruleus nuclei. Fourth order neurons were considered those labeled 4+ days PI and in regions more rostral, e.g., upstream, to the PVN including the lamina terminalis, organum vasculosum of lamina terminalis, subfornical organ, median preoptic nucleus, anteroventral periventricular nucleus, bed nucleus of stria terminalis, medial/lateral preoptic nucleus, suprachiasmatic nucleus, anterior hypothalamus, and lateral hypothalamus. Labeling in these regions was usually bilateral, and similar across all animals analyzed, except in one animal with labeled neurons in the primary motor area and visceral area of the insular cortex.

At 2 days PI, labeling in the nodose ganglia was absent, but present after 3–4+ days PI, whereas labeling in the NTS was seen at 4 days PI. NTS labeling suggests a direct (vagal) or indirect (spinal) pathway for afferent input or retrograde labeling from RVLM-projecting NTS neurons involved in sympathetic outflow. One study observed PRV-labeled neurons in the dorsal motor nucleus of the vagus (DMX), the parasympathetic nucleus for efferents traveling in the vagus nerve, 2.5 days PI (Cheng et al., 2022). PRV-labeled neurons in the DMX were chole acetyl transferase+ (ChAT+), suggesting these DMX PRV-labeled neurons are cholinergic parasympathetic efferents that innervate the kidney.

Labeled neurons were observed in DRG from T6-L3 with some labeling in contralateral DRG (∼1–3% labeled) 3–4+ days PI. Both renal denervation and dorsal root transection prior to renal injections attenuated labeling to the spinal cord, but still resulted in labeled ipsilateral DRG, indicating that PRV reached renal DRG neurons via both peripheral and central processes. Labeling in the dorsal horn of the spinal cord was more robust 4–5 days PI with laminae I and II showing labeling at day 4, and extending to laminae IV, V, VII, X, and dorsally to the central canal at 5 days. Schramm and colleagues (1993) reported labeling in dorsal horn neurons in the absence of DRG labeling (Schramm et al., 1993). Since PRV has a higher affinity for sympathetic neurons, labeled dorsal horn neurons without labeled DRG suggests retrograde labeling from sympathetic preganglionic neurons in the IML. This labeling raises the possibility of an intraspinal circuit for the regulation of renal function involving renal afferent terminations on dorsal horn interneurons or local second order neurons that synapse onto preganglionic IML efferents.

Tang et al. (2004) used PRV to identify the subpopulations of interneurons that are involved in modulating renal function via their connections to renal sympathetic preganglionic neurons (Tang et al., 2004). PRV-labeled interneurons were found in lamina II-V, VII, and X with the densest number in laminae VII. PRV-labeled interneurons were usually near PRV-labeled sympathetic preganglionic neurons suggesting a segmental organization and potential role in spinal sympathetic reflexes or reno-renal reflexes.

PRV tracing has been combined with immunostaining to identify neurochemical cell types of the PRV-labeled neurons: raphe nuclei (serotonin+ and SP+), RVLM and RVMM (phenylethanolamine-N-methyltransferase+, PNMT+), A5 cell group (tyrosine hydroxylase+, TH+), parvocellular division of the PVN (arginine-vasopressin+, AVP+) (Huang and Weiss, 1999). Each of these nuclei also had neuronal nitric oxide synthase+ (nNOS+) neurons, a sympathetic modulator, with the most in ventral parvocellular and posterior magnocellular subdivisions of the PVN, and most PRV/nNOS double-labelled cells in the dorsal parvocellular subdivision (Weiss et al., 2001). Serotonin+ and SP+ terminals were found close to dendrites of sympathetic preganglionic neurons in the IC and IML in the spinal cord, while PRV-labeled/nNOS+ neurons were found in the IML, IC and dorsal horn laminae I, III-V. The presence of neurons that express these peptides in close proximity to preganglionic sympathetic neurons highlights a potential role for these in modulating sympathetic renal activity and overall renal function.

Overall, PRV tracing provides additional evidence supporting the efferent innervation, i.e., regulation of sympathetic activity, to the kidney and underscores the relatively unknown pathway of renal afferent input to the brain. These findings highlight a number of additional regions that are likely involved in renal function. An important note, however, is that the PRV labeling in these brain nuclei does not distinguish between renal efferent and afferent circuitry, especially as post-injection time increases. Premotor neurons in central autonomic nuclei involved in sympathetic outflow are labeled from preganglionic sympathetic neurons, which in turn labeled higher order neurons, e.g., fourth order, in more rostral osmosensitive regions and potentially cortical areas. The regions mentioned in this review have since been identified and connected in several studies to be regulatory regions of interest in homeostatic processes, and so have a high likelihood of being involved in the processing pathway of renal afferent input.

## 3 Proposed central circuit processing of renal afferent input: an overview within the PVN

**FIGURE 2:**
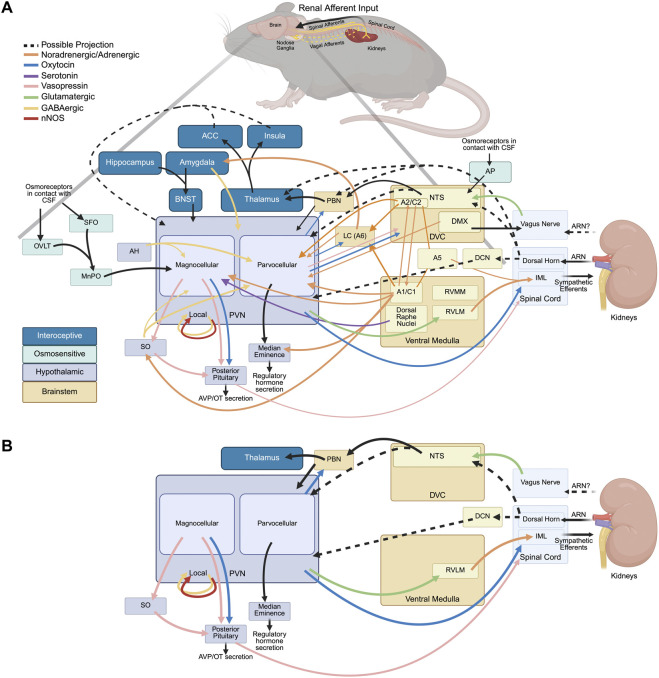
Connectivity of central autonomic regions likely involved in renal afferent input processing, organized in general reference to rodent brain neuroanatomy. Renal afferents enter the central nervous system via spinal afferents, and potentially via the vagal afferents. **(A)** Overview of connections between brain nuclei involved in renal afferent input processing. **(B)** Renal afferent input processing through the paraventricular nucleus of the hypothalamus (PVN). Inputs from renal afferents reach the nucleus of the solitary tract (NTS) and dorsal column nuclei (DCN) with further processing in the PVN through unknown intermediate stops. Renal afferent input can modulate activity in the PVN leading to changes in vasopressin release via the posterior pituitary gland and premotor sympathetic output to the interomediolateral cell column (IML) via the rostral ventrolateral medulla (RVLM). Abbreviations: A1/C1, noradrenergic/adrenergic cell group in ventrocaudal medulla; A2/C2, noradrenergic/adrenergic cell group near DVC; A5, noradrenergic cell group in caudal medulla; ACC, anterior cingulate cortex; AH, anterior hypothalamus; AP, area postrema; ARN, afferent renal nerve; AVP, arginine-vasopressin; BNST, bed nucleus of the stria terminalis; CSF, cerebrospinal fluid; DCN, dorsal column nuclei; DMX, dorsal motor nucleus of the vagus nerve; DVC, dorsal vagal complex; IML, interomediolateral cell column; LC/A6, locus coeruleus; MnPO, median preoptic nucleus; nNOS, neuronal nitric oxide synthase; NTS, nucleus of the solitary tract; OT, oxytocin; OVLT, organum vasculosum of the lamina terminalis; PBN, parabrachial nucleus; PVN, paraventricular nucleus of the hypothalamus; RVLM, rostroventrolateral medulla; RVMM, rostroventromedial medulla; SFO, subfornical organ; SO, supraoptic nucleus.

Figure 2A summarizes connectivity between brain nuclei that are likely involved in renal afferent input processing, and Figure 2B illustrates the processing of renal afferent input via the PVN. Calaresu and Ciriello initially identified single unit responses in the hypothalamus and medulla to electrical stimulation of renal afferent nerves (Ciriello and Calaresu, 1980; Calaresu and Ciriello, 1981). Xu et al. (2015) reported electrophysiological evidence of ipsilateral RVLM-projecting PVN neurons responsive to renal afferent nerve stimulation (Xu et al., 2015). These findings demonstrate that renal afferent nerve input modulates activity in the PVN, but through an unknown multi-synaptic pathway either via direct input to the NTS or dorsal column nuclei (Simon and Schramm, 1984; Wyss and Donovan, 1984; Knuepfer and Schramm, 1985) or indirect input first processed in the dorsal horn (Ciriello and Calaresu, 1983; Kuo et al., 1983; Knuepfer et al., 1988). The extensive anatomical characterization of the PVN has implicated it as a key integration and regulatory site for homeostatic control of body processes (Swanson and Sawchenko, 1980, 1983; Swanson et al., 1981; Sawchenko P. E. and Swanson L. W., 1982; Sawchenko P. E. and Swanson L. W., 1982; Van Den Pol, 1982). The complex organization of subnuclei and different neurochemical cell types provides the architecture for an integration hub that can handle simultaneous computations from the periphery, e.g., visceral input, and send responses to maintain homeostasis, e.g., effector output.

The PVN has two major components, the parvocellular and magnocellular divisions, that in total have eight distinct nuclei described by their rostro-caudal/dorso-ventral anatomical position (Swanson and Kuypers, 1980). The parvocellular subnuclei, five in total, express oxytocin (OT) and arginine-vasopressin (AVP). The medial parvocellular and periventricular subdivisions project to the median eminence, a region of the pituitary gland that secretes regulatory hormones into the circulation. OT outputs from the parvocellular division primarily project to the locus coeruleus (LC), parabrachial nucleus (PBN), dorsal vagal complex (DVC: containing DMX and NTS), and all levels of the spinal cord. Descending connections to the LC have reciprocal noradrenergic connections to the periventricular and medial parvocellular subdivisions, while the PBN, DMX, and NTS have more broad projections to the parvocellular subnuclei. Projections to the DVC are mostly from the medial or lateral subdivisions. OT parvocellular neurons provide greater input to the DVC compared to AVP parvocellular neurons (Swanson and Sawchenko, 1980). Most spinal cord-projecting parvocellular neurons are found in the dorsal (90% of neurons), medial, and lateral subdivisions. The IML in T9-L2 spinal segments receive a higher density of OT input with collaterals in the marginal zone, IC and dorsal central gray commissure (Swanson and Sawchenko, 1980; Zimmerman et al., 1984). Spinal AVP input from the lateral and ventromedial parvocellular divisions has also been identified (Zimmerman et al., 1984; Kolaj and Renaud, 1998; Hallbeck and Blomqvist, 1999). OT and AVP input to the preganglionic sympathetic neurons in the IML may have a role in modulating sympathetic activity involved in renal function. It has been proposed that spinal AVP input may be modulated by osmolality-induced sympathoexcitation via the PVN, however, two studies have shown opposing results (Antunes et al., 2006; Veitenheimer and Osborn, 2011). The magnocellular subnuclei, three in total, express OT in the anterior and medial subdivisions, and OT and AVP in the posterior subdivision. Magnocellular neurons are neurosecretory and release AVP and OT via axonal projections to the posterior pituitary gland into circulation. Some neurons in the posterior magnocellular nucleus have been found to project to the caudal brainstem nuclei and the spinal cord. Afferent input to the magnocellular division has been described in a review by Brown et al. (2013), highlighting the complexity of peripheral, central and local hypothalamic input onto magnocellular neurons (Brown et al., 2013). Overall, the parvocellular component is more involved in efferent output to the caudal brainstem and spinal cord compared to magnocellular neurons projecting to the posterior pituitary. Approximately 10% of cells in the PVN express somatostatin or TH and appear to project to the dorsomedial medulla or spinal cord. The role of these neurons is currently unknown.

Noradrenergic and adrenergic fibers have been found throughout all PVN nuclei originating from the brainstem, limbic system, visceral relay centers, and other hypothalamic nuclei (Swanson and Sawchenko, 1980; Swanson et al., 1981; Cunningham and Sawchenko, 1988). Nishi et al. (2017) reported that electrical stimulation of renal afferent nerves activated TH+ neurons in the RVLM and non-TH+ neurons in the NTS (Nishi et al., 2017). The parvocellular division of the PVN primarily receives noradrenergic input from A1 (caudal ventrolateral medulla), A2 (medial NTS), and A6 (near LC) cell groups, while the magnocellular division appears to only receive input from the A1 cell group. Visceral afferent input is known to travel primarily through the vagus nerve to synapse in the NTS. Afferent renal nerves also have direct (monosynaptic) input to the NTS and DCN via the spinal cord (Simon and Schramm, 1984; Wyss and Donovan, 1984; Knuepfer and Schramm, 1985). The direct projections from the A2 cell group in the NTS to an effector part of the PVN, i.e., parvocellular division, suggests that this catecholaminergic input may influence sympathetic outflow and maintain homeostasis. Noradrenergic fibers were more densely present near AVP+ nuclei in the PVN and supraoptic nucleus (SO), suggesting that catecholaminergic release, e.g., from DVC to PVN, on AVP-expressing neurons may reflect modulation of AVP release by visceral sensory input to the NTS. Additional evidence implicated catecholamine input to the PVN may have a role in modulating osmoreceptor activity near local capillaries and influence the movement of water. Changes to circumventricular organ osmoreceptors would impact the receptor sensitivity to solutes and likely result in efferent output to modify fluid homeostasis since osmoreceptor inputs are integrated in the PVN (Brown et al., 2013; McKinley et al., 2015). Since systemic fluid regulation is mediated by the kidneys, changes in solute concentration in the urine and blood would directly affect renal afferent chemosensitive input to the PVN which is integrated with osmoreceptor inputs from the cerebrospinal fluid to influence sympathetic activity and neurohumoral mechanisms, e.g., AVP release, in a feedback loop mechanism.

GABA has been estimated to be the most abundant neurotransmitter in the PVN comprising of approximately half of the synapsing boutons, with many of these inhibitory inputs arising from local GABAergic neurons (Decavel and Van Den Pol, 1990; Roland and Sawchenko, 1993). Interestingly, the magnocellular division had a 3× larger proportion of somatic GABAergic contacts compared to the parvocellular division. Moreover, individual GABAergic terminals were found to have synaptic contacts with multiple adjacent neurons, indicating a role for synchronized inhibition in a population of neurons. Alternatively, some PVN neurons were found to have multiple somatic contacts by the same GABAergic axon, suggesting tight regulation of the post-synaptic neuron. These observations suggest a major role for functional disinhibition within the hypothalamus, e.g., the activation of GABAergic inputs onto a GABAergic neuron would result in the disinhibition of its post-synaptic contacts. Evidence for tonic GABA inhibition of glutamatergic PVN neurons was provided by the effects of a GABA receptor antagonist infusion in the PVN, which resulted in an increased release of glutamate, leading to a higher renal sympathetic nerve activity, blood pressure, and heart rate (Li et al., 2006). Consistent with a sympathoexcitatory role of renal afferent input to the PVN under pathological conditions, dysregulation of PVN GABAergic signaling in a model of hypertension was attenuated by ablation of renal afferent nerves (Milanez et al., 2020). These findings highlight the importance of PVN GABAergic signaling in regulating sympathetic outflow from the PVN, and the potential PVN mechanisms of the anti-hypertensive effects of renal denervation.

nNOS also plays a prominent inhibitory role within the PVN. nNOS has been colocalized with PRV tracing in brain nuclei associated with sympathetic efferent output (Weiss et al., 2001). Microinjections of nNOS inhibitors into the PVN in rats resulted in increased renal sympathetic nerve discharge, arterial blood pressure, and heart rate (Zhang et al., 1997), indicating that under physiological conditions nNOS decreases sympathetic efferent output from the PVN. The effects of nNOS are mediated via GABA-dependent inhibition of spinally projecting PVN neurons (Zhang and Patel, 1998; Li, et al., 2002). A role of renal afferent input in regulating PVN nNOS was demonstrated by the observation that afferent renal denervation restored nNOS levels in the PVN of rats with chronic heart failure (Zheng et al., 2018).

Renal afferent input into the caudal brainstem has been identified, however, where and how these inputs are processed is still unclear. The PVN is a centralized nucleus that receives peripheral input for integration and sends efferent signals for autonomic and neurohumoral control of homeostasis. The intrinsic complexity of the PVN via its cytoarchitectural organization creates hubs for information integration and modulation, creating a system capable of parallel processing. The PVN receives direct input from the vagus nerve via the NTS and indirect input through the PBN. Visceral afferent signals ultimately result in effector signals that either engage sympathetic (RVLM to spinal cord) or parasympathetic (DMX to vagus nerve) circuitry to maintain homeostatic body processes. The precise pathway of higher order processing of visceral sensory input, including renal input, prior to or after integration in the PVN is complex and, so far, speculative. There is hope that these pathways will soon be elucidated with increasing interest in interoception (Evans et al., 2024), an understanding of how internal and external body sensation contribute to an overall body state, and with continued technological advancements.

## 4 Knowns, unknowns and opportunities

The clinical significance of afferent renal nerves has been highlighted by trials of catheter-based renal nerve ablation for drug-resistant hypertension. Independently of lowering blood pressure, this procedure has been found to reduce fasting plasma glucose, sleep apnea, cardiac arrhythmias, and muscle sympathetic nerve activity (Kiuchi et al., 2019). These effects can only be explained by afferent renal nerve ablation and suggest that hypertension-induced chronic increase in afferent renal nerve activity drives increased sympathetic activity to the kidneys, heart, and other organs (Schlaich et al., 2009; Osborn and Banek, 2018). In preclinical models of neurogenic hypertension, selective afferent renal denervation has been shown to reduce hypertension to the same extent as total (afferent and efferent) denervation (Banek et al., 2016, 2018, 2019; Ong et al., 2019; Lopes et al., 2020; Lauar et al., 2023, 2024; Baumann et al., 2024), highlighting the critical role of renal afferent nerves in perpetuating the pathophysiological changes contributing to hypertension. Clinically, there has been interest in translating the selective afferent renal denervation for treatment of drug-resistant hypertension. Large animal models, e.g., sheep, offer opportunities for the translational research required to parse out the long-term effects of afferent renal denervation (Booth et al., 2015; De La Cruz-Lynch et al., 2025). Identifying these long-term effects necessitates an increased understanding of renal afferent functions in homeostasis and pathophysiology. Therefore, there is a critical need for a thorough analysis of the functional properties and distribution of renal afferent nerves in order to harness the therapeutic potential of their manipulation through pharmacological or non-invasive neuromodulation approaches. Additional investigations into the presence of vagal afferent renal innervation may reveal other therapeutic targets, as vagal nerve stimulation for depression and epilepsy have been FDA approved and have had beneficial outcomes for patients (Nemeroff et al., 2006; O’Reardon et al., 2006; Milby et al., 2008).

The canonical pathway of renal afferent innervation begins with renal afferent cell bodies in the DRG. Projections from these DRG either synapse in the dorsal horn of the spinal cord between laminae I-II onto secondary neurons that project to the brainstem or project deeper synapsing in laminae IV-VII on interneurons and preganglionic sympathetic neurons likely involved in reno-renal reflexes (Ciriello and Calaresu, 1983; Kuo et al., 1983; Ferguson et al., 1986; Knuepfer et al., 1988; Weiss and Chowdhury, 1998). For some renal afferents, there are direct projections to the caudal brainstem, mostly localizing around the NTS or dorsal column nuclei (Simon and Schramm, 1984; Wyss and Donovan, 1984; Knuepfer and Schramm, 1985). Tracing and electrophysiological studies have been intertwined throughout the exploration of afferent innervation of the kidney and have provided complementary evidence for the anatomic investigations of renal afferent circuitry.

Within the kidney, renal afferents are most abundant in the renal pelvis (Reinecke and Forssmann, 1988; Ferguson and Bell, 1988; Marfurt and Echtenkamp, 1991; Burg et al., 1994). Despite a majority of tracer injections being targeted to the renal cortex, few studies have reported afferent fibers in the renal cortex (Reinecke and Forssmann, 1988; Marfurt and Echtenkamp, 1991; Ditting et al., 2009). Recently, renal cortical afferents have been quantified by their proximity to glomeruli (Tyshynsky et al., 2023), however, the presence of afferent fibers near different parts of the renal tubules has yet to be described.

Few early studies described labeling in the nodose ganglia suggesting vagal afferent innervation of the kidney (Gattone et al., 1986; Weiss and Chowdhury, 1998). However, there appear to be major inconsistencies of this labeling across studies compared to the consistency of renal afferent neuron labeling between T7-L2 DRG (Donovan et al., 1983; Norvell and Anderson, 1983; Ferguson et al., 1986; Gattone et al., 1986; Weiss and Chowdhury, 1998). The development of AAVs with better transduction in the kidney may provide a method by which these results can be reassessed, e.g., as reported by Cheng and colleagues (Cheng et al., 2022). This appears to be the only study that used AAV successfully in the kidney so far, and is the most recent to describe parasympathetic efferent innervation of the kidney, while evidence for vagal afferent innervation is still sparse (Zheng and Lawson, 1994; Ong et al., 2019).

Circuitry processing renal afferent input in the brain still have yet to be uncovered. A few studies have reported neuronal activation in central autonomic nuclei after electrical stimulation of renal afferent nerves (Ciriello and Calaresu, 1980; Calaresu and Ciriello, 1981; Xu et al., 2015; Nishi et al., 2017). General anatomic characterization of the PVN has implicated it as a region for integration and regulation of homeostatic and autonomic processes (Swanson and Sawchenko, 1983). Trans-synaptic retrograde labeling from the kidney has led to a more complete understanding of sympathetic outflow from the PVN to the kidney compared to the current understanding of renal afferent input processing in the brain (Ferguson et al., 1986; Schramm et al., 1993; Weiss and Chowdhury, 1998; Huang and Weiss, 1999). Additional studies are required to map the pathways processing input of renal afferent nerves beyond their central terminations in the spinal cord and brainstem. Functionally, the physiological mechano-, chemo-, or nociceptive stimuli encoded by renal afferent nerves and their impact on central autonomic circuits and homeostatic processes is still unclear.

Several different species have been used to anatomically investigate renal afferent fibers and their cell bodies. Researchers should be mindful of potential inter-species differences when extrapolating anatomical findings between model organisms, such as rats and mice (Price and Flores, 2007; Ong et al., 2019; Stocker and Sullivan, 2023). There is also ambiguity in distinguishing between differences in tracing methods used in different animal models and intrinsic differences in the species’ anatomy, e.g., extent of afferent arborization (Fazan et al., 2002). Since the majority of anatomical analysis of renal afferent nerves has been conducted in rats or larger species, there is currently a gap in our understanding of the mouse renal afferent innervation, for example, the relative abundance and overlap of CGRP-, SP-, and TRPV1-expressing renal DRG neurons or their central terminations. New viral tracing tools and transgenic techniques present opportunities to overcome this gap. For example, a transgenic approach that leverages the developmental expression of afferent markers has allowed mapping of the spatiotemporal innervation of the kidney during embryonic to adult mouse development (N’Guetta et al., 2024).

Physiologically there are renal afferent subtypes that respond to different types of stimuli, including mechanosensitive and chemosensitive (Niijima, 1971; Uchida et al., 1971; Recordati et al., 1978; Recordati et al., 1980, 1981; Kopp, 2015). The relationship between renal afferent functional response properties and neurochemical signatures is uncertain. Neuropeptide labeling in different regions of the kidney implies a rich diversity of afferent nerves that is not well defined (Kuo et al., 1984; Su et al., 1986; Reinecke and Forssmann, 1988; Ferguson and Bell, 1988; Knight et al., 1991; Burg et al., 1994; Zheng and Lawson, 1994; Ditting et al., 2009; Ong et al., 2019). While the distribution of labeled fibers in the kidney after tracer injection in the DRG resembles that of immunolabeled CGRP+ renal afferents (Marfurt and Echtenkamp, 1991), it also highlights the existence of other CGRP- neurochemical subtypes. Advances in understanding the diversity of cutaneous afferent innervation (Zheng et al., 2019; Sharma et al., 2020; Qi et al., 2024) offers a roadmap for the physiological, anatomical and transcriptional mapping of renal afferent nerves, which begins with single-cell RNA sequencing (scRNAseq).

Usoskin et al. (2015) provided one of the initial comprehensive scRNAseq references for the different neuronal subtypes in mouse lumbar DRG (Usoskin et al., 2015). They identify five broad transcriptomic classifications of DRG neurons with different numbers of subtypes and amounts of myelination. More recent transcriptomic studies have refined the classifications and generated mouse Cre-driver lines that allow selective tracing and functional analysis of transcriptional subtypes (Sharma et al., 2020; Qi et al., 2024). These studies have largely focused on the lumbar L3-L5 DRG, with the exception of Sharma and colleagues that highlights the different proportions of DRG neuron subtypes at different levels of the spinal cord (Sharma et al., 2020). While a great resource for those studying primary afferents innervating the skin and muscle, they leave many outstanding questions about the subtypes that innervate visceral tissues which receive both spinal and vagal afferent innervation. When considering the identified subtypes in these studies and the similar mechano- and chemosensitive responses that would be required of visceral afferents, notably likely to organ-specific endogenous stimuli, it can be assumed that many of the cutaneous DRG neuron subtypes likely overlap with those innervating the viscera. Any additional transcriptomic differences that may be cutaneous or visceral-specific have yet to be identified.

To date there are very few transcriptomic studies of the nodose ganglia, an area of research hopefully increasing in popularity due to interest in the vagal innervation of visceral organs and integration in interoceptive processes. A 2019 study (the earliest found at the time of this review) identified the transcriptomic and molecular specialization of vagal sensory neurons, e.g., jugular and nodose ganglia (Kupari et al., 2019). The researchers compared the vagal sensory neurons to a known DRG neuron subtype reference (Usoskin et al., 2015). They found that the jugular ganglia neurons most closely aligned with somatosensory neuron subtypes, while the nodose neuron subtypes represented distinctly different mechano- and chemosensitive subtypes, i.e., more visceral-specific afferent subtypes. With the relatively controversial evidence so far of renal vagal afferent innervation, it is still important to consider that some of these more visceral-specific subtypes may innervate or at least represent transcriptomic subtypes similar to renal primary afferent neurons.

Current studies have provided insight into the role of renal afferents by investigating their activity during pathophysiological conditions (Osborn et al., 2021; Evans et al., 2025). However, there is still a substantial gap in understanding the physiological functions of renal afferent nerves and the molecular mechanisms that enable them. Uncovering renal afferent transcriptional subtypes will unlock the ability to selectively trace and manipulate renal afferents, allowing for a more direct interrogation of their role within the kidney and how they contribute to physiology and pathophysiology.
